# Prognostic value of sinus CT scans in hematopoietic stem cell transplantation

**DOI:** 10.1590/S1808-86942010000500014

**Published:** 2015-10-22

**Authors:** Erica Ortiz, Érika Nakamura, Érika Nakamura, Cármino Antonio de Souza, Carlos Takahiro Chone, Afonso Celso Vigorito, Eulalia Sakano

**Affiliations:** 1 Physician, ENT specialist, master's degree in medical sciences - otorhinolaryngology at the FCM-UNICAMP. Collaborating otorhinolaryngologist in the Rhinology Unit, FCM-UNICAMP; 2 Physician, ENT specialist, resident in the Otorhinolaryngology Discipline, UNICAMP; 3 Medical resident, Otorhinolaryngology Discipline, UNICAMP. Otorhinolaryngologist; 4 Full professor at the Hemocentro, UNICAMP, and bone marrow transplantation at the HC-UNICAMP; 5 Professor Doutor, Head & Neck Surgery Otorhinolaryngology Discipline, UNICAMP; 6 Doctorate in hematology, medical hematologist and supervisor of the Bone Marrow Transplant Unit, Hemocentro/HC-Unicamp; 7 Doctorate in medical sciences - otorhinolaryngology. Physician of the Rhinology Unit, Otorhinolaryngology Discipline, FCM-UNICAMP. Otorhinolaryngology - Head & Neck Discipline/Rhinology Unit, Hospital das Clínicas - UNICAMP

**Keywords:** sinusitis, x-ray computed, tomography scanners.

## Abstract

**Abstract:**

Hematopoietic Stem Cell Transplant (HSCT) causes immunosuppression and predisposition to sinusitis. CT scans are complementary exams used in the diagnosis of sinusitis; however, its use in every patient is questionable.

**Aim:**

to check the usefulness of ordering a CT scan prior to HSCT and to study the relationship between anatomical variations and sinusitis.

**Method:**

prospective study in which we performed paranasal CT scans before and after HSCT, using the Lund and Mackay score.

**Results:**

77.5% and 61% of CT scans showed no evidence of sinus disease before and after HSCT. CT staging was not associated with sinusitis after HSCT. Anatomical variations were related to radiographic disease severity, but not to development of sinusitis after HSCT. There was no relation between pre-CT staging and sinusitis after BMT.

**Conclusion:**

CT scans are not useful for all patients before HSCT. Anatomical variation is not a predictive feature to sinusitis but it can determine its severity.

## INTRODUCTION

Hematopoietic stem-cell transplantation (HSCT) is a reality in tertiary hospitals at present. The number of transplants has increased gradually in Brazil during the past six years; over seven thousand transplants were carried out during this period. The number of HSCT in 2008 increased by 37% according to the National Transplant Unit of the Brazilian Ministry of Health.^1^

HSCT patients are previously immunosuppressed because of whole body chemotherapy or radiotherapy. They therefore are at a higher risk for viral, bacterial or fungal infection, in particular upper airway infections, because of direct contact with the environment.^2^, ^3^, ^4^, ^5^, ^6^, ^7^ Other complicating factors for respiratory infection are prolonged hospital stay, graft versus host disease, and corticosteroid therapy.^2^, ^3^, ^4^, ^5^, ^6^, ^7^ These patients have a 37% risk for developing rhinosinusitis after transplantation, compared to a 15% rate in patients with normal immune status.^3^,^8^

Several laboratory tests are required by the hematology unit before HSCT, including paranasal sinus radiography, done in all patients to investigate possible sinus disease. Some authors advocate doing computed tomography of the paranasal sinuses before transplantation in all patients, aiming at avoiding post-HSCT rhinosinusitis because of immunosuppression.^5^,^7^,^9^,^10^

According to the Brazilian rhinosinusitis guidelines, the diagnosis of this condition is not based only on paranasal sinus radiograms, which is insufficiently specific (79%) or sensitive (76%).^11^,^12^ At present, recommendations include a detailed clinical history and physical examination, and nasal endoscopy, which jointly enable a diagnosis of rhinosinusitis. Paranasal sinus computed tomography (CT) is done only if symptoms persist after treatment, or if the disease recurs or complicates.12 Thus, the physical examination and nasal endoscopy are effective for assessing patients before HSCT. However, some authors have defended carrying out paranasal sinus CT in all patients before HSCT, arguing that signs suggesting rhinosinusitis in this exam - and their intensity - are effective prognostic factors.^5^,^6^,^7^,^9^,^10^

Carrying out CT in all patients before transplantation may increase costs and the preparation time, as radiology units are under great demand for routine exams. Thus, the purpose of this study was to verify the need for paranasal sinus CT in all patients before HSCT and the relation between tomography findings of prior nasosinusal disease and the development of rhinosinusitis after HSCT.

## METHOD

A prospective pilot study was carried out in the Rhinology Unit of the Otorhinolaryngology Discipline. The institutional review board approved the study (protocol no. 088-2002). Thirty-one HSCT patients from the Bone Marrow Transplant Unit (2003-2004) were assessed. All patients underwent a clinical evaluation, nasal endoscopy, and paranasal sinus CT before and after HSCT. No patient had undergone nasal surgery before.

Patients with predisposing factors such as smoking, asthma, and nasal polyposis were excluded, as were patients in which CT was not done before or after transplantation.

The criteria of the Brazilian rhinosinusitis guidelines (2008) were applied.^12^ Accordingly, rhinosinusitis is characterized by nasal block, coughing, fever, facial pain, rhinorrhea with discharge, a purulent discharge in the middle meatus and/or sphenoethmoidal recess upon the nasal endoscopy, and/or sinus opacification on paranasal sinus CT.^12^

Anatomical changes were evaluated on CT (septal deviation, an altered unciform process, a bullous middle turbinate, a paradoxal middle turbinate, Haller cells), as were signs of rhinosinusitis (partial or complete opacification of paranasal sinuses).^13^, ^14^, ^15^ The Lund and Mackay classification was applied for the CT staging of rhinosinusitis.^13^ In this approach, each paranasal sinus is scored from 0 to 2 depending on the degree of opacification. Absence of changes is scored 0, partial opacification is scored 1, and complete opacifiation is scored 2. The osteomeatal complex is also scored depending on the degree of obstruction; no obstruction is scored 0, and obstruction is scored 2. Thus, a higher score corresponds to increased severity of rhinosinusitis - paranasal sinuses are more affected. The total scores ranged from 0 to 24 points for the right and left sides.

The relation between anatomical changes and/ or CT staging and the development of post-HSCT rhinosinusitis was analyzed. Fisher's test was applied in the statistical analysis of data.

## RESULTS

The sample for analysis comprised 31 HSCT patients - predominantly allogenic bone marrow transplants (90%). There were 19 females (61%) and 12 males (39%). The mean follow-up time for these patients was 12 months.

A pre-HSCT clinical diagnosis of rhinosinusitis was made in five patients (16.1%); this same diagnosis was made post-HSCT in 10 patients (32.2%), The maxillary sinus was the most frequent site, followed by the anterior ethmoid sinus (Table 1).

**Table 1:** Number of affected paranasal sinuses in pre- and post-HSCT computed tomography.

Paranasal sinus	Maxillary	Anterior ethmoid	Posterior ethmoid	Frontal	Sphenoid
Pre-HSCT	6 (75%)	1 (12.5%)	0	0	1 (12.5%)
Post-HSCT	13 (54%)	7 (29.2%)	1 (4.2%)	2 (8.4%)	1 (4.2%)

Only seven patients (22.5%) had prior CT staging over 1 for rhinosinusitis before HSCT; the same score was found in 12 patients (39%) after HSCT. Thus, 77.5% of CTs were within normal limits before bone marrow transplantation, and 61% were within normal limits after HSCT. Two (29%) of seven patients with positive rhinosinusitis staging before HSCT had no changes on CT after HSCT; two patients (29%) remained with the same staging score, and three patients (42%) had a worse score. Among patients with post-transplant rhinosinusitis, six (50%) had a CT stage over 2, without any change in the pre-HSCT CT.

Only six patients (19.4%) had anatomical changes or variants in the nasal cavity and/or paranasal sinuses, including septal deviation (3), bullous middle turbinate (3), and Haller cells (1).

Nasal and/or paranasal sinus anatomical variants were not associated significantly with a higher occurrence of pre- or post-HSCT rhinosinusitis (Table 2 and 3).

**Table 2:** Relation between anatomical alterations (AA) and the occurrence of rhinosinusitis (RS) before HSCT (p=0.96).

Rhinosinusitis	With RS	No RS	Total
Presence of AA	2	4	6
Absence of AA	3	22	25
Total	5	26	31

**Table 3:** Relation between anatomical alterations (AA) and rhinosinusitis (RS) after HSCT (p=0.932).

Rhinosinusitis	With RS	No RS	Total
Presence of AA	3	3	6
Absence of AA	7	18	25
Total	10	21	31

**Figure 1:**
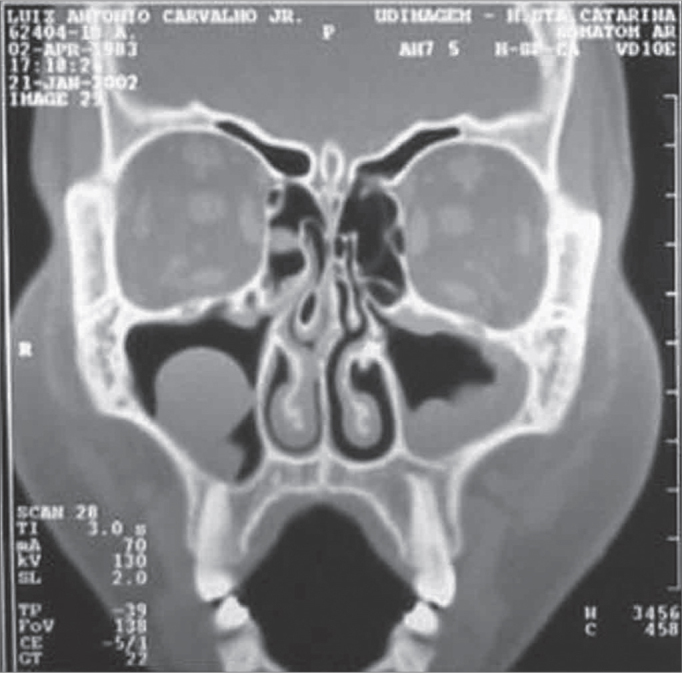
Paranasal sinus comupted tomography showing anatomical variants in a HSCT patient: bullous turbinate and septal deviation.

Anatomical changes were significantly associated with a higher post-transplant CT staging score for post-transplant rhinosinusitis (>2), which may be considered a complicating factor for post-transplant middle meati block (Fig. 1). Most of the group with anatomical changes - in relation to osteometal complex block - had a CT staging score of (Table 4).

**Table 4:** Relation between anatomical alterations (AA) and CT staging of rhinosinusitis after bone marrow transplantation (P=0.021).

Lund & Mackay score	Score (post)= 0	Score post >2	Total
Presence of AA	1	5	6
Absence of AA	18	7	25
To t al	19	12	31

The association between CT staging of paranasal sinuses before transplantation and rhinosinusitis after bone marrow transplantation was not significant (Table 5).

**Table 5:** Relation between CT staging for rhinosinusitis before HSCT (Pre Score) and the occurrence of rhinosinusitis after HSCT (p=0.128).

Rhinosinusitis	With RS	No RS	Total
Score = 0 pre-HSCT	6	18	24
Score > 1 pre-HSCT	4	3	7
Total	10	21	31

## DISCUSSION

A higher rate of post-HSCT rhinosinusitis is due to immunosuppression and to other aggravating factors such as prior chemotherapy and radiotherapy, viral infections, or graft versus host disease.^2^, ^3^, ^4^, ^5^, ^6^, ^7^ The published estimated rate of rhinosinusitis in transplant patients is 36.9%.^3^ The rate found in our study (32.2%) was roughly close to this estimate.

Notwithstanding the small number of patients in our study, a normal CT exam before HSCT was rather higher (77%) compared to other published results (51- 48%).^5^,^6^,^9^ Half of the patients in this study that presented post-HSCT rhinosinusitis had no prior CT findings or any clinical diagnosis of this condition. About 57% of patients with pre-transplantation CT findings had post-transplant rhinosinusitis, although there was not a significant correlation. Thompson et al.'s retrospective study also found no correlation between pre-transplantation CT findings and nasosinusal symptoms and post-transplant rhinosinusitis.^6^ Although this author's study had a larger sample - 100 pre-transplant patients during a 10-year period - only 64 post-HSCT paranasal sinus CTs were available for study.6 Shaw et al. studied 26 patients and correlated positive pre-transplantation CT findings with post-transplantation rhinosinusitis.^5^ The percentage rate of worsening of rhinosinusitis after bone marrow transplantation in previously affected patients (42%) was lower than that of Billings et al. (66.7%).^9^ Such differing results may be explained by the age range of study groups; the sample comprised only adults in Thompson's and our studies, and only children in Billings and Shaw's study. Thompson further explains this difference as due to pre-HSCT treatment of nasosinusal diseases; this was not clear in Billings and Shaw's study. All patients in the present study were treated before transplantation if they presented nasosinusal diseases.

Calculating the sample size was unnecessary, as this was a prospective pilot study to check the feasibility of carrying out paranasal sinus CT in all transplant candidates. Furthermore, the literature has no similar prospective studies corroborating CT before HSCT.

Most HSCT studies have small samples because

of the paucity of transplantations. Thus, a large sample is only possible in multicentric studies. Within our study period (2003 to 2004) there were 79 allogenic HSCTs at the Bone Marrow Transplantation Unit, Hemocentro/HC-UNICAMP. The mean annual allogenic transplantation rate at our Unit from 2002 to 2004 was 40 transplants.

A lack of correlation between anatomical variants and the occurrence of rhinosinusitis before and after transplantation is in line with published studies on chronic rhinosinusitis in patients with a normal immune status.^14^,^15^ Thus, surgery before HSCT in patients with anatomical variants to prevent post-transplantation rhinosinusitis is not indicated. There was, however, a correlation between anatomical variants and post-HSCT worsening of rhinosinusitis. Larger samples may clarify this correlation.

Paranasal sinus CT in all HSCT-treated patients appears to be unnecessary because no significant correlation was found between positive nasosinusal CT findings and the occurrence of post-transplantation rhinosinusitis. A few authors have suggested carrying out paranasal sinus CT before transplantation in all patients, arguing that the severity of CT findings before HSCT would correlate with the occurrence of rhinosinusitis after HSCT.^5^,^9^

Paranasal sinus CT might be done only in HSCT candidates with clinical and endoscopic findings suggesting rhinosinusal disease.

## CONCLUSION

Although the sample was small, given the paucity of transplants, paranasal sinus CT before HSCT appears not to be necessary in all patients; the exception is evidence of anterior nasosinusal disease.

Rhinosinusitis demonstrated in pre-transplant CT showing altered sinuses, as seen in this study, may be related with worsening of the rhinosinusitis after transplantation. Nasosinusal anatomical variants appear to be associated with more severe rhinosinusitis after bone marrow transplantation.
